# A WSi–WSiN–Pt Metallization Scheme for Silicon Carbide-Based High Temperature Microsystems

**DOI:** 10.3390/mi7100193

**Published:** 2016-10-20

**Authors:** Ha-Duong Ngo, Biswajit Mukhopadhyay, Piotr Mackowiak, Kevin Kröhnert, Oswin Ehrmann, Klaus-Dieter Lang

**Affiliations:** 1 Center of Microperipheric Technologies, Fraunhofer Institute IZM, Berlin 13355, Germany; Biswajit.Mukhopadhyay@izm.fraunhofer.de (B.M.); Piotr.Macokowiak@izm.fraunhofer.de (P.M.); Kevin.Kroehnert@izm.fraunhofer.de (K.K.); Oswin.Ehrmann@izm.fraunhofer.de (O.E.); kdlang@izm.fraunhofer.de (K.-D.L.); 2 University of Applied Sciences, FB I, Microsystems Engineering, Berlin 12459, Germany; 3 Microsensor and Actuator Technology, Technical University Berlin, Berlin 10623, Germany

**Keywords:** microelectromechanical system (MEMS), SiC-based microsystems, sensors for harsh environment, sensors for high temperature

## Abstract

In this paper, we present and discuss our new WSi–WSiN–Pt metallization scheme for SiC-based microsystems for applications in harsh environments. Stoichiometric material WSi was selected as contact material for SiC. The diffusion barrier material WSiN was deposited from the same target as the contact material in order to limit the number of different chemical elements in the scheme. Our scheme was kept as simple as possible regarding the number of layers and chemical elements. Our scheme shows very good long-term stability and suitability for SiC-based microsystems. The experimental evaluation concept used here includes a combination of physical, electrical, and mechanical analysis techniques. This combined advance is necessary since modern physical analysis techniques still offer only limited sensitivity for detecting minimal changes in the metallization scheme.

## 1. Introduction

In recent years, there has been an increasing demand for high temperature electronics, operating in harsh environments and at temperatures up to 500 °C. Various key industrial sectors such as petroleum & gas, geothermal logging, nuclear power, and the automotive and aerospace industries require materials that maintain reliability under such hostile conditions. Table 1 shows an overview of applications and temperature ranges.

One of the crucial features for successful device performance is the availability of a suitable long-term stable high temperature metallization scheme, typically including a diffusion barrier layer, which prevents a metallurgical reaction between metallization and semiconductor substrate, a contact layer and an interconnect layer. Ternary amorphous metallic thin films such as Ti–Si–N [1,2,3,4], Mo–Si–N [5,6], Zr–Si–N [7,8,9,10], Ta–Si–N [11,12,13,14,15,16], and W–Si–N [17,18,19,20,21,22,23] have received considerable attention for high temperature diffusion barrier applications. These materials have shown to be chemically inert against reactions with gold [4,11,24], silver [11], copper [12,13,14,16,17,20], aluminum [18,25], and platinum [26,27,28] metallization at elevated temperatures, thereby providing the low diffusivities required for a diffusion barrier. Moreover, their amorphous structure sustains even at temperatures above 800 °C [15,20,21], preventing the formation of grain boundaries which act as rapid diffusion paths.

However, most annealing experiments published to date have been limited to rather short annealing times of typically 30–90 min. This is sufficient for applications such as copper ultra large scale integration (ULSI) interconnects [5,13,17,22], dynamic random-access memory (DRAM) applications [29,30], and other devices that require short-term high temperature processing, but tells us little about the actual long-term high temperature stability of the metallization schemes presented.

Silicon carbide is considered to be the most promising semiconductor for the above-mentioned high-temperature and harsh-environment applications, as it features a unique combination of physical, electrical, mechanical, and chemical properties. This material is extremely hard and robust with high thermal stability. It has a wide bandgap, which allows operation at high temperatures and in high radiation environments.

In this work, we have investigated layered contact structures consisting of a 200 nm WSi_2_ contact layer, a 200 nm W–Si–N diffusion barrier, and 150 nm Pt top metallization for silicon carbide-based sensors. A systematic search for suitable materials for each of the three functional layers is extremely difficult. First of all, materials must be characterized and investigated down to a near-atomic level in order to understand the solid-state reactions and electronic properties of clusters involved in the contact formation and interdiffusion [31].

Stoichiometric layer of tungsten silicide (WSi_2_) was chosen as contact layer of a 6H–SiC substrate. This material is known for its low electrical resistivity and excellent thermal and chemical stability. Deposition and patterning techniques for the silicide layer are well developed, and low-resistivity ohmic contact to SiC have been demonstrated. Works with other transition metal silicides such as TaSi_2_ are ongoing and will be published later.

The barrier layer is WSiN. It is focused on the thermal stability of amorphous W–Si–N thin films and their performance as diffusion barriers between WSi_2_ and platinum. Samples were annealed at 500 °C for up to 1000 h in N_2_ ambient conditions and examined via X-ray diffraction (XRD) and Auger electron spectroscopy (AES) analysis.

Platinum is known for its excellent high temperature stability and could be used as top metallization for the chip and bond interconnect material for micro welding. In order to get the best adhesion of Pt, sputtering at room temperature was chosen for the platinum deposition process.

## 2. Experimental Details

### 2.1. Chip Layout and Materials

**Figure 1:**
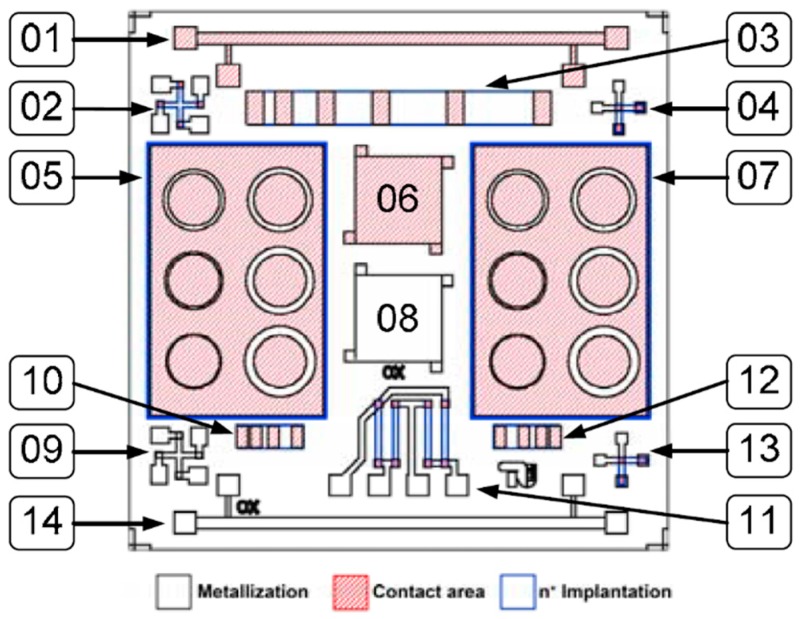
Test chip layout for electrical tests of the contact resistivity in the SiC–WSi–WSiN–Pt metallization system. The upper bone structure (structure 1) and the van-der-Pauw structures (structure 6) are used to characterize the metallization in contact with the substrate. Bone structures (structures 8 and 14) are used to measure the metallization sheet resistance. The Greek cross structure (structure 2) can be used to determine the semiconductor sheet resistance. The Greek cross structure (structure 9) can be used to determine the sheet resistance of the metallization stack. The structure 11 consists of 4 resistors is commonly used in a sensor device. The linear TLM structures (3, 10 and 12) and circular TLM (5 and 10) and two cross-bridge Kelvin resistors (4 and 13) are used to measure the specific contact resistivity.

The test chip layout is shown in Figure 1. There are two bone structures and two square van-der-Pauw structures. The upper van-der-Pauw structures are designed so that the metallization is in contact with the substrate. Those can be used to characterize the metallization in contact with the SiC substrate. In the lower structures, the metallization is insulated from semiconductor substrate, so that structures can be used to measure the metallization’s sheet resistance without influence of the subjacent substrate. The upper Greek cross structure can be used to determine the semiconductor sheet resistance, while the lower one can be used to determine the sheet resistance of the metallization stack. The layout consists of four implanted resistors and mimics a Wheatstone-bridge measurement circuit, as is normally used in sensor devices. Three linear transmission-line matrix (TLM) test structures, two circular TLM structures, and two cross-bridge Kelvin resistor structures have been used for measuring the specific contact resistivity. In addition, the I–V characteristics of the metal semiconductor contact can be measured using the contact pads of the linear TLM structure. The I–V curve indicates whether an ohmic contact has been formed, and changes in the curve’s slope indicate changes in the metallization’s sheet resistance or contact resistivity.

Silicon wafers were used in this work (to test thermal stability) were purchased from Okmetic Ltd., Vantaa, Finland. The 100 mm material was (100) oriented, with an *n*-type with a typical resistivity of 1–10 Ω·cm and a thickness of 525 µm. The substrates were oxidized to obtain 100 nm SiO_2_ as insulation before deposition of metallization.

The 6H–SiC wafers used had a thickness of 250 µm and were oriented 8° off-axis. The silicon face was prepared using Epi-ready polishing technique. The substrates were nitrogen-doped with a typical resistivity of 70 mΩ·cm. Two epitaxial layers were deposited on the polished face of the wafers. First, 12 µm ± 2 µm of *p*-SiC, doped with an aluminum concentration of 3 × 10^17^ cm^−3^, were deposited, followed by 1.3 µm ± 0.2 µm of *n*-SiC doped with a nitrogen concentration of 5 × 10^18^ cm^−3^. The epitaxial layers improve the surface quality of the substrates by closing micropipe defects and create an abrupt *pn* junction for the later realization of piezoresistors by mesa etching.

### 2.2. Investigation of Ohmic Contact between WSi and SiC

Tungsten silicide contact layer was deposited from a Cerac 200 mm high-density, hot-pressed WSi_2.3_ composite target with a purity of 2N5 (99.5%). The layer thickness was adjusted to 150 nm. The resistivity was measured to be 480 µΩ·cm.

A contact formation step was performed in order to achieve a low resistivity ohmic contact and transform the amourphous silicide layer into their respective high-temperature phase. The wafers were annealed in a Xerion Xreact 1000 rapid thermal processing system (Xerion Advanced Heating GmbH, Freiberg, Germany) for 1 min at 1100 °C in a high purity argon atmosphere. The resistivity of the annealed layer was measured to be 50 µΩ·cm.

**Figure 2:**
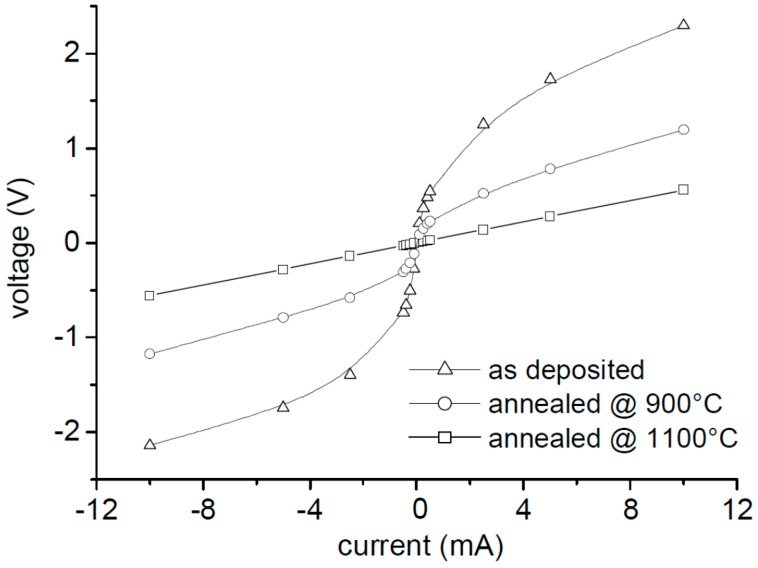
I–V characteristics of samples from SiC-wafer after rapid thermal annealing (RTA) at different temperatures.

The I–V curves of the samples, annealed at different temperatures, are depicted in Figure 2 below.

**Figure 3:**
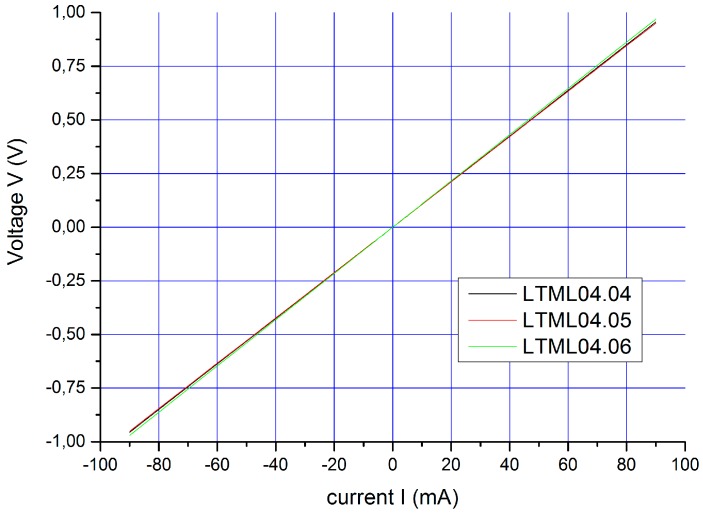
I–V curve measured at a linear transmission line model (LTLM)-structure on a SiC-wafer (200 nm WSi_1.8_ on 6H–SiC), annealed for 1 min at 1100 °C.

As deposited, the WSi–SiC system shows a strong non-linear behavior. With increasing annealing temperature, the I–V characteristic becomes increasingly linear. For an annealing time of 1 min at 1100 °C, we reached a perfectly ohmic behavior over a broad current range, as illustrated in Figure 3.

All tested chips show a perfectly linear I–V behavior in a wide range of ±100 mA. This confirms our results and demonstrates that the WSi contact layer forms ohmic contacts on 6H–SiC.

The contact resistivity was measured using the Kelvin test structure. The minimum contact resistivity was measured to be 6.2 × 10^−4^ Ω·cm^2^, and the mean value is 6.6 × 10^−4^ Ω·cm^2^. This value again confirms previous results.

### 2.3. Investigation of Thermal Stability of the WSiN Barrier Layer

For our experiments, unpatterned 4 inch (100)-Si wafers (*n*-type, phosphorous, 1–10 Ω·cm) were used as substrate material. The substrates were oxidized by dry thermal oxidation at 1000 °C in O_2_ ambience to create 100 nm of SiO_2_. WSi_2_ contact layers with a thickness of 200 nm were sputter-deposited in a Leybold Heraeus Z660 Load Lock Sputtering System (Leybold GmbH, Cologne, Germany) from a high purity WSi_2.3_ composite target. The thin films were DC magnetron-sputtered at 1.5 kW forward power using krypton gas at a pressure of 5 × 10^−3^ mbar. A rapid thermal processing system Xerion Xreact 1000 was then used to anneal the samples for 1 min at 1000 °C in a high purity argon flow. W–Si–N barrier layers with a thickness of 200 nm were deposited by reactive sputtering using the same WSi_2.3_ target as that of the contact layers. Barrier layers with three different compositions were direct current (DC) magnetron-sputtered at 1.0 kW forward power using gas mixtures of 10 sccm N_2_ + 80 sccm Ar, 5 sccm N_2_ + 85 sccm Ar, and 2 sccm N_2_ + 88 sccm Ar, respectively. Finally, 150 nm of platinum were sputter-deposited at room temperature.

For all further experiments, the wafers were cut into pieces of about 20 mm × 30 mm. The samples were annealed for up to 1000 h in a Centrotherm vertical tube furnace at 500 °C in a 8 slm high purity N_2_ flow. After 24 h, 100 h, 500 h, and 1000 h, dedicated samples were removed from the furnace and examined via optical microscopy and scanning electron microscopy (SEM) to study the surface morphology of the metallization scheme. XRD analysis was used in order to characterize the microstructure and thermal stability of the W–Si–N barrier films. XRD measurements were performed at room temperature using CoKα radiation, a step width of 0.05°, and scattering angles of 2θ = 20–80°. Auger electron spectroscopy (AES) was employed to determine the film composition and study the extent of thin film interdiffusion between the three metallization layers.

#### 2.3.1. Elemental Composition of W–Si–N

Three sets of W–Si–N samples with different nitrogen amounts were sputtered-deposited using various nitrogen partial flow rates. Table 2 shows the deposition conditions and atomic composition of the investigated W–Si–N barrier layers as determined via AES analysis. The samples are labelled with letters A–C for abbreviation. The as-deposited layers have an atomic composition of W_27_Si_70_N_3_, W_25_Si_69_N_5_, and W_25_Si_66_N_9_, respectively. By increasing the nitrogen partial flow from 2 to 10 sccm, the nitrogen content in the deposited W–Si–N layers increased from 2.5 to 9 atom %. At the same time, the Si–W atomic ratio decreased from 2.7 to 2.38. All samples furthermore contain 1.4 atom % of argon and traces of oxygen (below 0.5 atom %) and carbon (below 0.2 atom %), which is in the rage of the respective detection limit.

#### 2.3.2. Microstructure of WSiN

**Figure 4:**
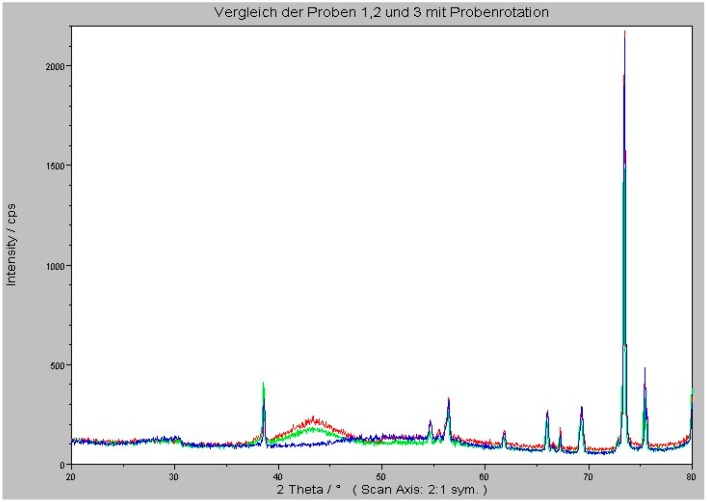
XRD analysis of samples after 100 h annealing at 500 °C in N_2_.

The thermal stability of the WSiN thin films was investigated. Samples with WSiN layers on oxidized Si substrates were processed and analyzed via XRD. Figure 4 shows the XRD spectrums for films deposited on SiO_2_, annealed at 500 °C in nitrogen ambient conditions for 100 h. The upper curve shows the spectrum of the WSiN layer and the lower curve the Si substrate, measured on the backside of the sample.

All films present amorphous structures in the as-deposited and annealed conditions when analyzed by XRD diffraction. Only a broad peak centered at 2θ = 43° was detected in the XRD patterns.

The broad peak at 2θ = 43° is indicative of an amorphous layer. No crystalline compounds were found in the deposited layers. All peaks originate from the Si substrate. This composition is thermally stable at temperatures up to 500 °C. This is in agreement with the literature [19]. Thermal stability, in the amorphous phase, of the WSiN films is crucial for the use of this material as a diffusion barrier. Our results show that all three WSiN films are thermally stable for a long time. Crystallization leads to the formation of grain boundaries, which act as rapid diffusion paths and lead to barrier failure.

**Figure 5:**
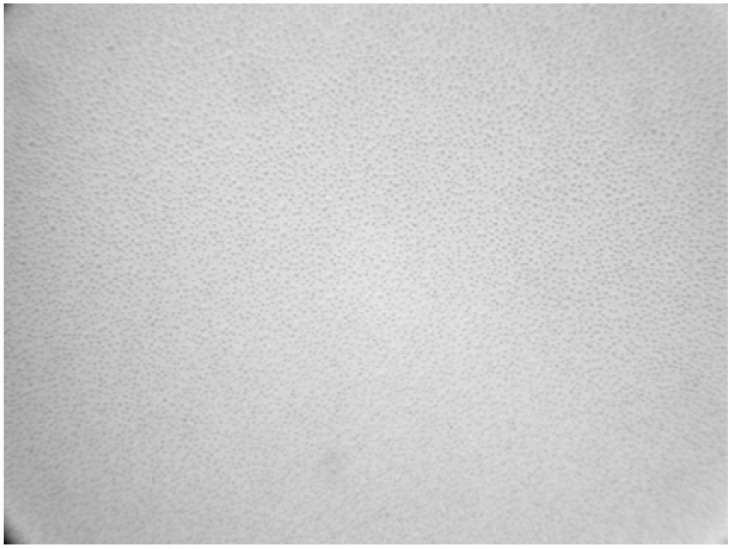
WSi–WSiN–Pt metallization after 100 h of annealing at 500 °C in air (magnification 500×).

Samples with platinum deposited by evaporation at 350 °C shows small blisters after annealing for 100 h at 500 °C in air as depicted in Figure 5.

### 2.4. Characterization of the New Metallization Scheme

**Figure 6:**
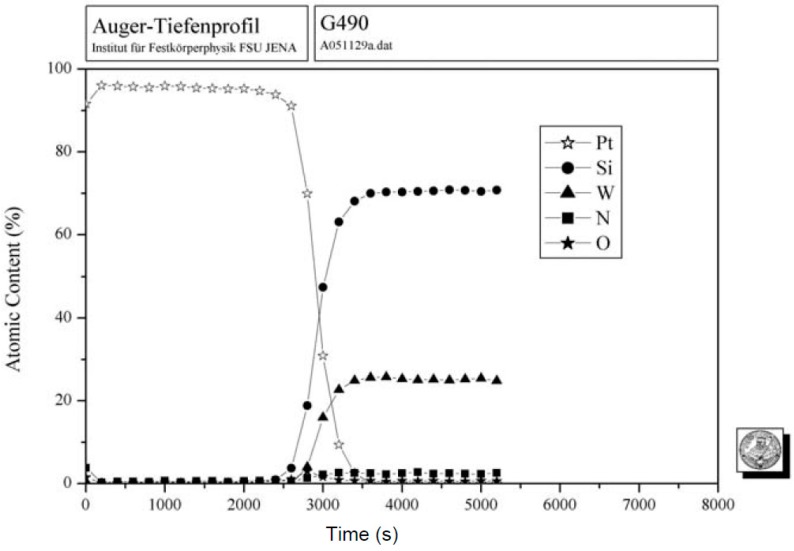
Auger electron spectroscopy (AES) analysis of a sample with a WSiN barrier layer and 150 nm Pt top metallization, annealed for 1050 h at 500 °C in N_2_ ambient conditions.

In order to test the barrier capabilities of our new metallization scheme, a sample was annealed for 1050 h at 500 °C in N_2_ ambient conditions for AES analysis. The result is very promising. As depicted in Figure 6, there is no platinum diffusion into the WSiN barrier layer.

Silicon carbide test chips were processed using the complete, optimized WSi–WSiN–Pt metallization system. Chips were annealed at 500 °C in N_2_ ambient conditions for 70 h, 120 h, and 410 h, respectively, in order to investigate the change in contact resistivity upon annealing. After an initial increase of 1.42%, the contact resistivity remains almost constant. The maximum variation after the initial anneal is as low as 0.14%.

## 3. Conclusions

In this work, new high-temperature-stable metallization concept for SiC-based microsystems was developed. The metallization concept comprises the choice of materials as well as the setup. After processing, a multilayer, multi-element metallization system is generally not in state of thermodynamic equilibrium. Subsequent long-term high-temperature annealing provides the activation energy for material redistribution and chemical reactions towards an energetically favorable state. These mechanisms cause the electrical contact to become unstable. The advance in this study was to minimize the driving force for such reactions within the metallization stack. Stoichiometric material WSi was selected as contact material for SiC. The diffusion barrier material WSiN was deposited from the same target as the contact material in order to limit the number of different chemical elements in the scheme.

WSi_2_ contact layers with a thickness of 150 nm were sputter deposited in a Leybold Heraeus Z660 Load Lock Sputtering System from a high purity WSi_2.3_ composite target. A rapid thermal processing system Xerion Xreact 1000 was then used to anneal the samples for 1 min at 1000 °C in a high-purity argon flow. This is necessary to transform the amorphous WSi layers into their stable tetragonal phase, yielding low layer resistivity, ohmic linear electrical behaviour, low intrinsic stress, and high temperature stability.

The thermal stability of WSiN thin films was investigated. All films present amorphous structures in the as deposited and annealed condition, when analyzed via XRD. Only a broad peak centered at 2θ = 43° is detected in the XRD patterns. The broad peak at 2θ = 43° is indicative of an amorphous layer. No crystalline compounds were found in the deposited layers. All peaks come from the Si substrate. This composition is thermally stable at temperatures up to 500 °C.

Our scheme was kept as simple as possible regarding the number of layers and chemical elements. Our scheme shows very good long-term stability and suitability for SiC-based microsystems.

The experimental evaluation concept used here includes a combination of physical, electrical, and mechanical analysis techniques. This combined advance is necessary since modern physical analysis techniques still only offer limited sensitivity for detecting minimal changes in the metallization scheme.

## Figures and Tables

**Table 1:** High temperature applications and associated temperature ranges.

Application	Measuring Site	Temperature (°C)
Power Plant	Power Engine, Turbine, Waste Gas Cleaning	500–600 °C 800 °C 300–400 °C
Logging	Drilling Head	250–300 °C
Plastic Injection	Plasticization Area or at Nozzle	150–500 °C
Petroleum	Reactor	200–1000 °C
Medicine	Instruments Sterilization	150–300 °C
Automotive	Combustion Engine	150–2000 °C

**Table 2:** Atomic composition of ternary metal silicon nitride barrier layers tested in this study as determined by AES analysis.

Target	Me–Si Ratio	N_2_ Partial Pressure (%)	Resistivity (µΩ·cm)	Atomic Composition	Me–Si Ratio in Film
WSi_2.3_	1:2.3	2.2	479.4	W_27_Si_70_N_3_	1:2.6
WSi_2.3_	1:2.3	5.6	529.6	W_25_Si_70_N_5_	1:2.8
WSi_2.3_	1:2.3	11.1	595	W_25_Si_66_N_9_	1:2.6
